# Effects of a play-based approach on psychosocial variables in federated long- and middle-distance athletes

**DOI:** 10.3389/fpsyg.2024.1481417

**Published:** 2024-12-17

**Authors:** Alfonso Valero-Valenzuela, Luz Amalia Hoyos Cuartas, Diego Andrés Heredia-León, Patxi León-Guereño

**Affiliations:** ^1^Health, Physical Activity and Education Research Group, Physical Activity and Sport Department, Sport Sciences Faculty, University of Murcia, Murcia, Spain; ^2^Faculty of Physical Education, National Pedagogical University, Bogotá, Colombia; ^3^Academic Unit of Education, Catholic University of Cuenca, Cuenca, Ecuador; ^4^Department of Physical Activity and Sport Science, Faculty of Education and Sport, University of Deusto, Gipuzkoa, Spain

**Keywords:** interpersonal teaching style, track and field, self-determination theory, motivation, play activities

## Abstract

**Introduction:**

Considering the theory of self-determination and its relationship with human motivation and the play-based approach (PBA), a training program is implemented in young middle and long-distance running athletes with play activities (simple tasks with rules in a ludic mood). The objectives were (1) to analyze the effects of a PBA on the autonomy support interpersonal teaching style (ASITS) perceived by athletes, the psychological variables of satisfaction of basic psychological needs, motivation, enjoyment and intention to continue practicing athletics and depending on sex, and (2) know the athletes' perception of this methodology after the intervention.

**Method:**

Quasi- experimental design with a sample of 50 athletes (27 women and 23 men) with 17.22 average of age and federated at the regional level.

**Instruments:**

Autonomy Support Scale (ASS), Psychological Need Satisfaction in Exercise Scale (PNSE), Behavior Regulation in Sports Questionnaire (BRSQ), Measure of Intention to be Physically Active (MIPA) and Intrinsic Satisfaction in Sports Questionnaire (ISSQ).

**Results:**

Significant changes were identified in favor of the experimental group compared to the control group in ASITS and autonomous motivation. Over time, only the experimental group showed positive changes in the ASITS variable, self-determination index, autonomous motivation, and intention to continue practicing athletics. The women in the experimental group presented higher values in the self-determination index, autonomous motivation and intention to continue practicing athletics, while the women in the control group only in the ASITS variable in the pre-test. The young people who practiced playing during the warm-up reported high levels of fun and motivation, and interest in the activity, as well as concern about being prepared for the main part of the session.

**Discussion:**

It is suggested to athletic trainers to use a PBA in athletics groups at a regional level to promote an ASITS and its positive consequences at both a psychosocial and cognitive level, although new studies are required, and of longer duration to be able to contrast these findings and their effects on athletic performance.

## 1 Introduction

From an anthropological perspective, play has occupied a predominant place in all human communities, from indigenous peoples to the present. In this sense, playing is considered an element of the culture of all civilizations and an educational activity for people of different ages (Blanchard and Cheska, 1986; Coakley, 2021).

In this way, there is a tacit agreement that relates play to learning in infants (Pyle and Danniels, 2017; Kerlinger and Howard, 2022). However, new educational trends call for the development of research that evaluates the effectiveness of play in the learning of schoolchildren and other population groups (Danniels and Pyle, 2018; Bubikova-Moan et al., 2019). In this sense, the use of play as a means of learning has been incorporated into teacher training processes with the purpose of making its application efficient in the classroom (Díaz-Varela and Wright, 2019; Ntshangas and Venketsamy, 2022).

With respect to the teaching of modern sports, since its appearance in the 18th century it is evident to recognize an evolution throughout the last half of the 20th century and throughout the 21st century, from teaching models based on sports technique to alternative models that offer other roles to teachers, coaches and/or athletes. However, in this wide range of pedagogical models, play and games (henceforth, “game” will refer to games that work on improving tactics) have always had a place, of greater or lesser relevance, in accordance with the learning theories and the pedagogical models that support the theories (Hoyos, 2024; Mitchell et al., 2020; Ribas et al., 2023; Böke and Aygün, 2024).

In the path of cognitive development through games, the systematic review carried out by Gabbett et al. (2009) showed that the use of games for team sports causes improvements in the cognitive skills of youth athletes related to decision-making and the execution of skills autonomously, compared to other models that use repetitive technical instruction.

In more recent studies, the meta-analysis by Böke and Aygün (2024) revealed that the use of game-based models in school sports produced notable improvements in multidimensional domains such as critical thinking, motivation and skill expertise, especially in the context of dynamic and unpredictable game situations. Recent studies reveal the virtues of using this approach in sports initiation processes. The results of the study by Atiq et al. (2017), with beginner soccer players between 8 and 12 years old, found significant improvements in basic techniques with the use of play.

In summary, in recent decades there has been a consensus statement that unifies the different initiatives that use games for educational purposes in sport, under the name game-based approach to refer to the initiatives that use sports games as a strategy to form reflective, creative, intelligent and skilled children and young people. This approach promotes the development of metacognitive processes in athletes (Gambles and Gutierrez, 2023).

It is important to note that in general, the game-based approach has been used mainly in team sports, as shown in the literature review by Kinnerk et al. (2018) and in the study by O'Connor et al. (2020). The characteristics of the model have greater affinity with the nature of team sports.

However, initiatives in individual sports such as athletics have started to appear. Although initially called a game-based approach, they are closer to play activities (simple tasks with rules in a ludic mood) than games with a decision-making intention, because they have little or nothing to do with tactical content, being a more accurate term play-based approach (PBA). For example, the study by Peraza et al. (2018) demonstrates that the incorporation of play produces a positive impact to “achieve harmony in the development of technical skills, development of physical abilities and logical skills” in athletics athletes (p. 296). Along the same lines, the study by Triansyah et al. (2019) found that, from the perception of adolescents of an athletics club, the activities implemented optimized the learning of their running techniques.

Regarding research with child and youth athletes who implement the guide for mini-athletics proposed by the IAAF, the study by Blatsis et al. (2016) identified that the approach produced improvements in the level of motivation of the athletes, such as was evidenced through a questionnaire that evaluated self-determination and identity, finding an improvement in the performance of preadolescents in field and track events.

Research has made progress in establishing relationships between the use of these models and the psychological development of athletes. With this purpose, the systematic review of 17 studies carried out by Pérez-González et al. (2019), related to the use of models based on autonomy support by physical education teachers, found that these interventions have positive effects on motivation of students and in the satisfaction of basic psychological needs, being consistent with the study by Valero-Valenzuela et al. (2021), which found that the satisfaction of basic psychological needs is a positive predictor of self-determined motivation and physical self-concept of students of physical education.

The satisfaction of psychological needs and motivation have also been studied in relation to the level of future expectations and the levels of personal and social responsibility in physical education students by Manzano-Sánchez et al. (2019), finding that students with high future expectations have more autonomous motivation and greater satisfaction of their basic psychological needs, positively affecting the school climate.

In athletics, Manzano-Sánchez et al. (2020) studied the motivation of amateur runners in Spain regarding training patterns and sex, determining that men have greater task-oriented motivation, while in female athletes this motivation is greater in ego and sports performance. Starting from the theoretical foundations that argue the virtues of the use of the play in teaching and training processes in individual sports, it is necessary to understand how coaches use a PBA for the development of athletes, especially in individual sports such as athletics. Moreover, in the specific context of Ecuador, It was considered very important to evaluate the intention to be physically active in this age range because the practice of sports is generally interrupted with the beginning of higher education, since in Ecuador many students do not have access to a public university and have to work to pay for their studies (Heredia-León et al., 2023).

Based on this background, it is justified to carry out this quasi-experimental study in the sports field, which has the objective to analyze the effects of the implementation of a PBA in young athletes at the regional level on different psychological variables following the self-determination theory and the possible differences depending on sex, as well as knowing the perceptions that these athletes have about the use of the PBA and its usefulness for their preparation in the athletics events they practice. As a hypothesis, it is proposed that boys and girls who were taught through the PBA would report: (a) better results in the satisfaction of the different psychological variables and (b) better athletes' perceptions of the PBA in the preparation in the athletics events.

## 2 Materials and methods

### 2.1 Design

Experimental design (Thyer, 2012) of repeated measures was carried out with two intervention groups, one called the control group and the other experimental group.

### 2.2 Participants

In the present study, the sample was initially composed of 60 Ecuadorian athletes federated in athletics, specialized in medium distance and long distance at a regional level, belonging to the provincial sports federation of Azuay and the provincial sports federation of Cañar. After applying the exclusion criteria, the sample had 50 athletes (23 men and 27 women); with ages between 13 and 23 years (*M* = 17.22, SD = 3.40). The control group (*n* = 9) was made up of five men and four women (*M* = 18.55, SD = 2.45), while the experimental group (*n* = 41) was distributed in 18 men and 23 women (*M* = 16.93, SD = 3.46). The exclusion criteria for the final selection of the sample were not having attended 80% of the training sessions carried out and also not having completely responded to all the questionnaires implemented before and after the intervention. Questionnaires that were not answered in their entirety were eliminated. The type of sampling carried out was non-probabilistic, the athletes and coaches of the federations were intentionally selected for accessibility and convenience; and due to logistical constraints related to the distance of the federations and accessibility for both coaches and athletes, a random sampling method was impossible to employ. All participants were middle class.

In addition, a total of 3 athletics coaches belonging to the two provincial federations were part of the sample. The coach of the control group had a degree in Physical Activity and Sports Pedagogy and had 10 years of experience teaching athletics. The second coach, who taught part of the experimental group, was a sports training specialist, with 15 years of experience. The third coach who taught sessions to the rest of the experimental group had training in Educational Sciences with a mention in Physical Culture, with 20 years of experience teaching athletics.

### 2.3 Instruments

#### 2.3.1 Support for autonomy

Autonomy support was assessed using the Autonomy Support Scale (ASS) of Moreno-Murcia et al. (2020). It uses 11 items in which participants respond concerning the interpersonal style of the trainer in the practices aimed at supporting autonomy (eg, “Offers different ways of performing a certain task”). The previous statement used was: “In my training, my coach…”. It consists of a Likert-type scale with five response options, from (1) Certainly not to (5) Certainly yes. The internal consistency coefficients showed a Cronbach's alpha value = 0.81 and 0.82 for the pre-test and post-test respectively.

#### 2.3.2 Basic psychological needs satisfaction

Psychological Need Satisfaction in Exercise Scale (PNSE) by Wilson et al. (2006) was used, validated in the Spanish context by Moreno-Murcia et al. (2011). The PNSE uses 18 items, six to assess each of the needs: competence (eg, “I have confidence to do the most challenging exercises”), autonomy (eg, “I believe I can make decisions regarding my exercise program”) and relationship with others (eg, “I feel a camaraderie with my classmates because we exercise for the same reason”). The previous statement was “In my training…” and the answers were collected on a Likert-type scale, whose score range ranges from 1 (False) to 6 (True). Internal consistency revealed an α value for competence of 0.84 and 0.81 in pre-test and post-test, for autonomy of 0.68 and 0.71, and for relationship with others of 0.68 and 0.70. In addition, the index of psychological mediators (IPM) was developed by calculating the average of the three factors in a single dimension.

#### 2.3.3 Autonomous motivation

The factors that make up autonomous motivation from the Behavior Regulation in Sports Questionnaire (BRSQ) by Lonsdale et al. (2008), were used validated in Spanish by Moreno-Murcia et al. (2011). Six factors of four items each were used that measure general intrinsic motivation (eg, “Because I find it pleasant”), intrinsic motivation about knowledge (eg, “Because I enjoy learning new techniques”), intrinsic stimulation motivation (eg, “Because of the pleasure it gives me when I am totally dedicated to this sport”), intrinsic achievement motivation (eg, “Because it gives me satisfaction when I strive to achieve my goals”), integrated regulation (eg, “Because practicing this sport is part of who I am”), the identified regulation (eg, “Because it is a very good way to learn things that can be very useful in my daily life”). The previous statement was: “I participate in this sport…”. A seven-point Likert-type scale was used ranging from 1 (Very false) to 7 (Very true).

To analyze the data, following Williams et al. (1996) the four dimensions of intrinsic motivation, integrated regulation and identified regulation were unified into a single dimension called autonomous motivation. The reliability of the variables for the Ecuadorian athletes was α = 0.81 and 0.80 for the intrinsic motivation of knowledge; α = 0.79 and 0.70 for intrinsic stimulation motivation; α = 0.78 and 0.64 for intrinsic achievement motivation; α = 0.78 and 0.66 for the identified regulation; for the unified factor autonomous motivation it was α = 0.92 and 0.75.

#### 2.3.4 Future intention to be physically active

The questionnaire called “Measurement of intention to be physically active” (MIPA) by Hein et al. (2004) and validated in the Spanish context (Moreno et al., 2007) was used. This questionnaire is made up of five items (eg, “After I finish high school, I would like to stay physically active”). The previous statement was: “Regarding your intention to practice some physical-sports activity…”. The responses were closed with a Likert-type scale: totally disagree (1) to totally agree (5). The reliability value was α = 0.68 and 0.70, in the pre-test and post-test respectively.

#### 2.3.5 Sports satisfaction

Intrinsic satisfaction in sport: the Intrinsic Satisfaction in Sport Questionnaire (ISSQ) by Duda and Nicholls (1992) was used in its Spanish version (Balaguer et al., 1997). The questionnaire consists of eight items divided into two scales that measure Fun (five items) and Boredom (three items). The previous statement was: “Indicate your degree of disagreement or agreement with the following statements, referring to your athletic training…”. An example of a satisfying/fun item was (eg, “I usually find athletics interesting”) and a boring item was (eg, “When I practice athletics, I usually get bored”). The responses were collected on a 5-point Likert-type scale: ranging from strongly disagree (1) to strongly agree (5). The internal consistency analysis was for fun α = 0.69 in both the pre-test and the post-test.

#### 2.3.6 Interviews

The research objectives were taken as a starting point for the collection information. The lead researcher drafted a first version of the questions, which were then reviewed by the other authors of the study for clarity and accuracy. In addition, two colleagues who were experts in the field of qualitative studies were asked to review the questions to ensure they were clear, relevant, and aligned with the interview objectives.

Two semi-structured group interviews were carried out (Aguirre, 1995; Taylor and Bogdan, 1992), one with each federation with questions directed to the athletes in the experimental group with an approximate duration of 5 min. The athletes were chosen at random, a boy and a girl, and they were also asked to voluntarily choose another boy and another girl to participate together in the group interview, to gather opinions on the PBA and find out how it affects the different psychosocial variables of the study. The questions dealt with the general impression that this methodology caused in them with the question: What did you think of the new training methodology?, the aspects that most attracted you to your practice, what did you like the most?, and finally if this new approach could provoke greater adherence to the practice of athletics with the question: Does using this methodology motivate you to continue practicing this sporting discipline? The interviews were carried out at the end of the last training in a place away from the rest of the group. An adequate amount of time was allowed for the athletes to be fully rested, and the assessment was conducted in an outdoor environment, comfortable for the athletes and free of interruptions.

### 2.4 Procedure

This research had the approval of the Ethics Committee of the University of Murcia (3023/2020). Next, permission was requested from the Provincial Sports Federations of Azuay and Cañar—Ecuador. Contact was established with the managers and coaches responsible for the participating federations to inform them of the objectives and request their collaboration. The underage athletes were asked for written authorization from their parents, guardians or legal representatives. Once the relevant informed consent was obtained so that the athletes could participate in the study, they were informed how to complete the questionnaires and resolve any doubts that may arise during the process. The instruments were applied in person in the pre- and post-test of the experimental and control groups, by one of the researchers to explain the objective of the study, the questionnaires were administered at the beginning of the training, the anonymity of the responses was reported. The questionnaire administration time was ~15 min.

### 2.5 Intervention program

**Figure 1:**
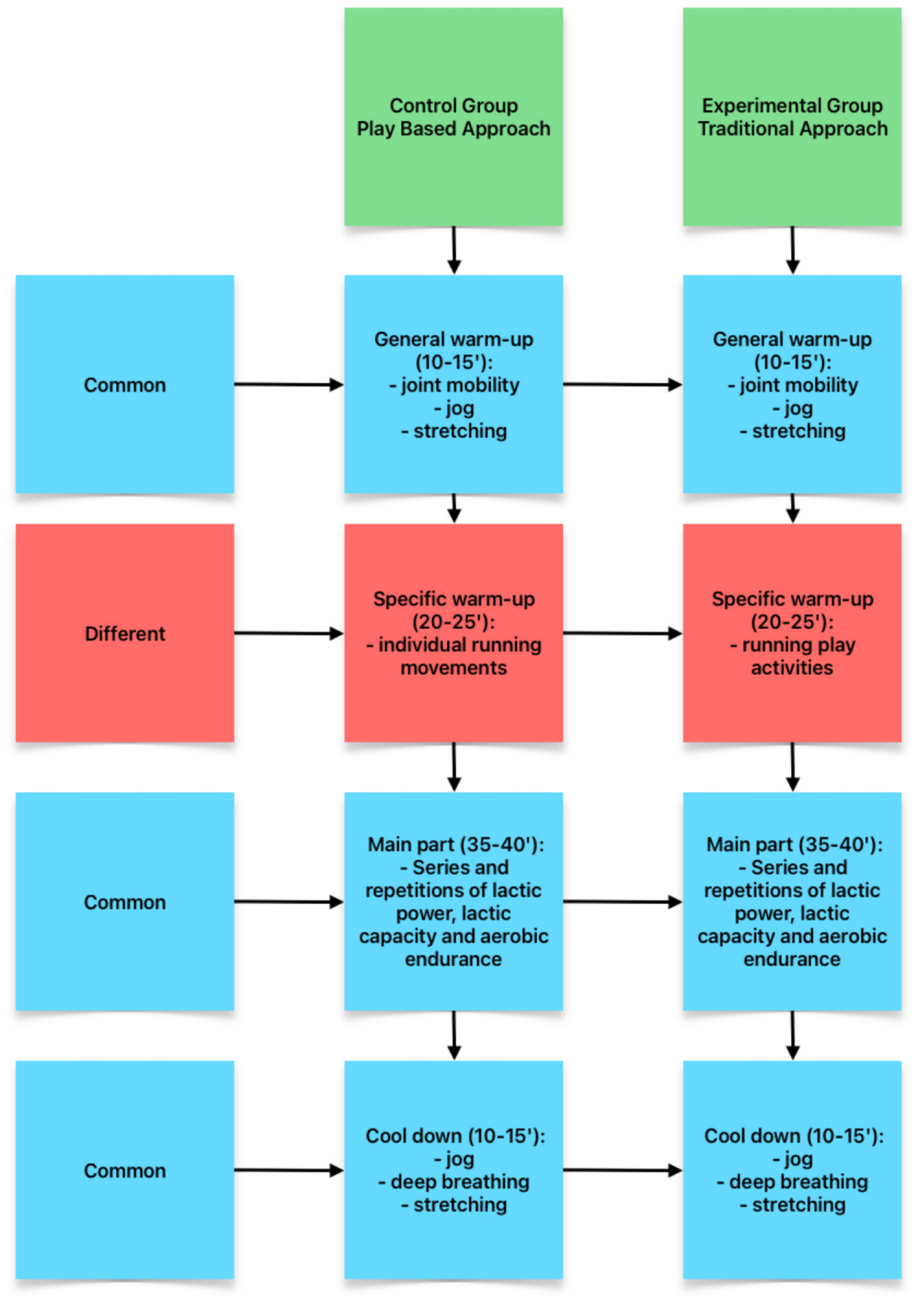
Main differences between Play Based Approach and Traditional Approach in this study.

In the experimental group, an intervention program was executed during the specific part of the warm-up based on play activities related to some of the athletics disciplines, with a duration of 8 weeks where the athletes developed activities as described in Table 1 (twice a week). In all sessions of both the experimental group and the control group, a general warm-up consisting of joint mobility activities, a jog of 5–10 min in total and stretching exercises was carried out (Figure 1). Later, for the experimental group, within the specific warm-up, play activities were carried out mainly linked to the races (Table 1; Figure 1) of 20–25 min, once the warm-up activities were completed, the athletes carried out the main part before calming down with their usual training according to the planning of each coach (Table 1; Figure 1).

**Table 1:** Description of the sessions implemented in the experimental and control groups.

**Session**	**Common in both groups**			**Experimental group**	**Control group**
	**General warm-up (10–15^′^)**	**Main part of the session (35–40^′^)**	**Return to calm (10–15^′^)**	**Specific warm-up: Play-based approach (20–25^′^)**	**Specific warm-up: Conventional approach (20–25^′^)**
1	In all sessions the warm-up consisted of: Joint mobility exercises: • Up and down head movements flexing and extending the neck • Circular head movements • Raise your shoulders and move them back and forth • Stretch your arms crosswise and make circular movements back and forth • Flex and straighten the elbows • Wrist movements performing circles and dorsal and palmar flexions • Rotating movements of the torso with the hands on the waist from one side to the other • Lateral inclinations of the trunk with the opposite arm stretched above the head • Arm raises and flexion behind the head • Leg exercises raising and carrying forwards and backwards, first one and then the other • Flexion and extension of the knees • Squats • Circular ankle rotation exercises Continue moderate running 10 min Stretching exercises 5 min	4 sets of repetitions of 200, 300 and 400 m with a pause of 1 min between repetitions and 3 min between sets	In all sessions the return to calm consisted of: Slow jog 5 min Deep breathing exercises • Inhale deeply through your nose, hold your breath for a few seconds, and then exhale slowly through your mouth. Repeat this process several times Static stretching exercises with the purpose of helping the body recover for the next session, for example: • Stretch for upper trapezius. Slowly pull your head to the right with your hand and lower your left shoulder, as if you wanted to extend your arm toward the floor, at the same time. Hold the stretch for the required time and then return to the starting position slowly. Breathe fluidly, do not hold your breath. Then switch sides • Standing oblique abdominal stretch arm overhead. Slowly bend your body from your waist toward your left side until you feel the stretch in your right oblique muscles. Exhale during the movement. Hold the stretch for the required time and then return to the starting position progressively. Do it with the other side. Can't hold the air • Standing forearm stretch. Pull on the back of your right hand to flex your wrist. Hold the stretch for the required time and then return to the starting position. Then change the arm • Shoulder stretch with arm in horizontal adduction. Slowly pull your elbow toward your chest until you feel the stretch in your deltoid. Hold the stretch for the required time and then return to the starting position. Then change the arm • Dorsal and lumbar vertebral stretch. Lie on your back with your feet flat on the floor. Your knees should be at $90^{\underline{\text{o}}}$. Place your hands on the floor next to your hips. Slowly raise your legs followed by your hips and trunk and try to reach the ground with your feet. The head should end between the legs. Hold the stretch for the required time and then return to the starting position • Supine knee to chest stretch. Lie on your back with your feet flat on the floor. Your knees should be at $90^{\underline{\text{o}}}$. Grab your right knee with your hands and slowly pull it toward your chest. Keep your left leg always extended. Hold the stretch for the required time and then return to the starting position. Then switch sides	Hurdle race It is divided into teams, a distance of ~40 m marked by cones is indicated. In the first part, the 40-m flat race is carried out and, on the return, once around the cone, the second part is executed, which is the passage over the fences, a total of 4 fences separated every 6 m. The play activity begins at the teacher's signal, first the flat race is carried out and on the return the hurdle race, a baton will be used that must be given to each participant, the team that completes the shortest time possible when all the athletes have run is the winner	In all sessions the specific warm-up consisted of: Specific exercises: • Walking with emphasis on the base of the metatarsus • Short jumps on the base of the metatarsus • Low skipping • Medium skipping • Skipping high • Skipping with the right leg (unilateral) • Skipping with the left leg (unilateral) • Heels to glutes. • Russian steps or Russian skipping • Pedaling with one leg • Pedaling with both legs • Continuous alternating jumps with maximum stride width • Vertical jumps raising knees and alternating legs • Three progressive runs or accelerations of 60 m
2		Continuous running for 30 min with a heart rate between 140 and 160 beats per minute		Formula One It is divided into teams, marking a route 80 m long, starting with a role on a mat, then executing a 20-m race, then performing zig-zag movements on a route marked by flags (10 m), continuing a 20-m race, perform a passage over 3 hurdles (10 m) and conclude with a 20-m race. A baton will be used that must be handed over to each participant. The team that completes the shortest time possible when all the athletes have run is the winner	
3		5 repetitions of 800 m with a 3-min break		Javelin throw for children It is performed from a 5-m approach area, after a short approach run, the participant throws the javelin toward the throwing area from the boundary line. Each participant has two attempts. Each throw is measured at $90^{\underline{\text{o}}}$ (right angle) from the limit line and is recorded at intervals of 25 cm (the largest number being taken when the fall occurs between the lines). The best of each team member's two attempts counts toward the team's final score	
4		Continuous running for 40 min with a heart rate of approximately 140 beats per minute		Endurance race Each team must run around a 150-m track, from a given starting point. Each team member -tries to run around the track as many times as possible in 8 min. The starting order is for all teams at the same time (whistle or shot, etc.) Each team member receives a card or something similar after completing a lap of the track. After 7 min the last minute is announced with another whistle or gunshot. After 8 min, the end of the race is indicated with a final signal After finishing, all participants hand all the cards to the assistant who counts them for the score. Only complete turns are counted; those that are not complete are ignored. The team with the greatest number of cards wins	
5		One repetition of 1,000 m, pause of 2 min, 15 repetitions of 200 m with pause of 100 m of active jogging		Round trip relay race Distances are marked with cones (short 10 m, long 25 m or long 50 m). The team members form a line behind the starting point. Once the signal is issued, the first member moves, goes around the cone, and returns to hand the baton to the second participant and forms his team's line. The second player cannot move until the first player receives the token and so on. The play activities alternate between different distances (short, medium or long). The team that finishes in the shortest time by organizing the line in the way it was initially formed wins	
6		Continuous running for 30 min with a heart rate between 140 and 160 beats per minute		Speed race The distance to be traveled is marked with cones, which can be between 10, 20 or 30 m. The first members of each team participate in groups. The exit will be alternated, for example, high exit, sitting, lying on the back, lying on the stomach, skipping in one's own place, etc. The participant who touches the cone is the one who awards a point to his team, the team with the greatest number of points wins	
7		2 sets of repetitions of 600, 800 and 1000 m with a pause of 4 min between sets and 2 min between repetitions		Rope jumping The participant stands with feet parallel in the starting position holding the jump rope behind his body with both hands. When the command is given, the rope is brought in front of the head and down in front of the body and the participant jumps over it. The cyclic process is repeated as many times as possible for 15 seconds with the purpose of reaching the greatest number of jumps. Each child has two attempts. The best result of each team member is considered for the final team score	
8		Continuous running for 40 min with a heart rate between 140 and 160 beats per minute		Javelin throw The throw at the target is carried out with an approach run of 5 m. A rod is located about 2.5 m high; the target area on the ground at a distance of 2.5 m from the rod. The chosen object is thrown onto the rod. The participant throws from a selected distance. Each participant has three attempts to hit the target with the thrown object. In each attempt, the participant can choose to throw from any of the four lines: 5 m, 6 m, 7 m, or 8 m away from the rod. The greater the distance, the higher the score. Hitting the target area—or at least on its edges—is considered a valid attempt. Points are recorded for each success (throws from 5 m = 2 points, 6 m = 3 points, 7 m = 4 points, 8 m = 5 points). If the object is thrown on the rod but misses the target, it is considered 1 point. Each participant has three attempts, the sum of these is considered for the team's total score	

The coaches of the experimental group received an initial training of 6 h (three times in a week, each day 2 h) in the PBA. The course covered content linked to the use of played activities as a means of learning, how to adapt playing to teach athletic disciplines, including keys for teaching them and examples for putting them into practice.

Meanwhile, in the control group, the instructors did not receive any specific training and continued to carry out the usual warm-up of their sports federation aimed at getting ready for the main part of their daily planning (Table 1). The specific warm-up consisted of individual, repetitive and isolated running exercises to improve running technique with the purpose of avoiding possible injuries that may occur during training such as low, medium or high skipping (Table 1; Figure 1).

For the general warm-up, the main part of the session and cool down were common to both groups. Specifically, the main part of the session consisted of series and repetitions of lactic power, lactic capacity and aerobic endurance work depending on the moment of preparation in which the athletes were (Table 1; Figure 1) and the return to calm that included a 5-min passive jog, deep breathing exercises and static stretching exercises with the purpose of helping the body recover for the next session (10–15′ in total). The total time spent in each session was ~1 h and 30 min.

Psychosocial variables were measured before and after the application of the program to verify the effectiveness of the program on these variables. The intervention took place during the usual training hours of the participating provincial federations.

### 2.6 Implementation fidelity

Following Hastie and Casey (Hastie and Casey, 2014), research should provide detailed validation of the intervention program based on models or strategies. A self-questionnaire based on the features of each approach detailed in Table 1, was developed to assess the coaches performance in the implementation of the sessions. The replies from control and experimental group coaches were reviewed by two expert judges. In addition, a checklist was prepared by these two expert judges (two doctors in sports sciences and athletics trainers with more than 10 years of experience as athletics instructors) to identify what type of activities they carried out in the specific warm-up part of the different sessions, distinguishing between: (1) assimilation exercises focused on improving technique, (2) application exercises or focused on physical condition, (3) play activities linked to the technical gesture of the discipline, (4) play activities similar to the athletic discipline, (5) motivational type feedback, (6) technical type feedback. From the sessions filmed in both the CG and the EG, the items were evaluated every 5 min of recording, so that if the trainer did what was mentioned in the item, it was marked with a “1” and if he did not do it with a “0.” At the end of the session, the percentage of appearance of each item was calculated.

For the analysis of the sessions, an external observer (Graduate in Sports Sciences) who was not involved in the research, was trained by the researchers of this study to record the performance of the coaches in relation to the items described above. The training process had the following sequence (Wright and Craig, 2011): (1) explanation and clarification of the meaning of each of the items of the instrument, different types of example situations were given so that they could be clearly distinguished); (2) the external observer and one of the main researchers viewed two complete sessions where the PBA was applied; (3) sharing of observation results by observers for the unification of criteria; (4) intra-observer reliability of 80% guaranteed to begin the analysis of the sessions. Total agreement (TA) was calculated by total agreements (TA) divided by agreements (A) plus disagreements (D): (AT = TA/A + D) (García-López et al., 2012).

### 2.7 Data analysis

A reliability analysis was carried out on all the scales and then the Mahalanobis distance was used to detect and eliminate those outliers or those that did not follow a logical pattern in the set of variables. In addition, the values of asymmetry and kurtosis were analyzed, with < 2 and < 7 respectively being considered appropriate (Curran et al., 1996). After eliminating nine participants who did not meet these requirements, the Cronbach's alpha reliability analysis of the different scales was carried out again, finally counting on the total sample of 50 participants. The reliability coefficients revealed values above 0.70, a criterion considered acceptable for psychological domain scales (Nunnally, 1978). Few alpha coefficients fell in the range between 0.66 and 0.70, an acceptable value for authors such as Sturmey et al. (2005).

A MANOVA (2 × 2 × 2 repeated measures analysis), was carried out on the 10 variables obtained from the different questionnaires, where the intra-subject factor was called Time (with two levels: pre-test and post-test) and as the inter-subject factor Group was considered (with two levels: control and experimental). Additionally, the inter-subject factor sex (two levels: male and female) and age were added as covariates, since it was found that they could have a significant effect on the measured variables. An analysis of the residuals revealed non-compliance with the normality hypothesis of some variables, so it was decided to also carry out the analyzes using non-parametric tests. The results obtained with both procedures were very similar, so the results of the nonparametric tests were not included for brevity. The implementation fidelity was analyzed using the *U* Man–Whitney non-parametric test, due to none of the variables revealed compliance with the normality hypothesis (Shapiro–Wilks). The statistical package IBM SPSS 25.0 (New York: USA) was used for the analysis.

For the analysis of the interviews, one of the researchers led the procedure and a second one overviewed it. Before starting the analysis, the textual quotes by the monitors from the two blocks that included the nine questions were extracted literally, to then choose the most relevant ones and carry out a synthesis of them. One of these researchers began to read and re-read the interview transcripts. This initial familiarization step allowed the researchers to identify recurring patterns and relevant aspects in the responses. These quotes were included for analysis in the so-called “codes” or “substantiated” that were based on the common characteristics of the extracts (eg, positive points of the model) to determine which elements had the most recurrence when responding to the questions as carried out by other studies (Seale, 1999) following Seale's sequence (Thyer, 2012) for data collection. This researcher highlighted text segments that represented key aspects related to the participants' experiences. In the next phase, the search for themes began, grouping the codes into thematic categories that captured the shared experiences of the participants. The emerging themes were reviewed for internal coherence and refined to ensure that they faithfully reflected the perceptions of the athletes. Finally, these themes were defined and named in an interpretive and reflexive manner, seeking an authentic and meaningful representation of the qualitative data, and establishing clear connections with the quantitative results of the study.

## 3 Results

### 3.1 Strategies used by teachers

With the aim of finding out the degree of implementation of the different methodological strategies linked to the PBA in one group and another, these were evaluated based on the checklist prepared by experts. Table 2 presents the values extracted based on the group, obtaining significant differences in the items: the coach provides motivational information to increase the participation of young people and provides technical information (items 6 and 7; *p* < 0.005). In all cases, values were higher for the experimental group, except for item 2 (physical conditioning exercises are performed). Furthermore, considering the effect of size, this item obtains a very large effect (*d* > 0.8). The rest of the items with a very large effect are items 4 and 5, play activities similar to the athletic events are played and play activities not linked to the technical gesture or the event are played.

**Table 2:** Evaluation of implementation fidelity.

	**Cluster experimental (*****N*** = **18)**		**Cluster control (*****N*** = **6)**		**U Mann-Whitney**	**Cohen's D**
	***M***	**SD**	***M***	**SD**	***p***	***r***
1. Technique exercises are performed	31.6	32.70	26.3	22.59	0.871	0.17
2. Physical fitness exercises are performed	73.4	33.79	100	0.00	0.077	0.89
3. Play activities linked to the technical gesture of the event are played	22.3	33.89	0.00	0.00	0.251	0.75
4. Play activities similar to the athletic event are played	23.6	30.83	0.00	0.00	0.119	0.87
5. Play activities not linked to the technical gesture, or the event are played	14.8	21.1	0.00	0.00	0.177	0.80
6. The coach provides motivational information (to increase the participation of young people)	90.3	24.5	0.00	0.00	< 0.001^*^	4.20
7. The coach provides technical information	53.2	35.1	8.33	20.40	0.006^*^	1.38

M, mean; SD, standard deviation; r, effect size.

^*^p < 0.005.

### 3.2 Effects of the program on athletes

To assess the effects of the intervention program in each group, a repeated measures MANOVA was carried out at a multivariate level. The results show that there are only significant differences at the inter-subject level for the Group factor [Wilks' Lambda = 0.606, *F*_(10, 36)_ = 2.336, *p* = 0.030] and for the Age factor [Wilks' Lambda = 0.533, *F*_(10.36)_ = 3.156, *p* = 0.005].

Subsequently, the results were analyzed at the univariate level showing that at the inter-subject level, there are significant differences for the Group factor, for the satisfaction of the basic needs, for relationship (*p* = 0.007) and fun (*p* = 0.026), for the Age factor, for the satisfaction of the need, basic relationship (*p* = 0.004) IPM (*p* = 0.005) and autonomous motivation (*p* = 0.015). For the Group and Sex interaction, there are also significant differences in the interpersonal style variable of autonomy support (*p* = 0.020), autonomous motivation (*p* = 0.029) and intention to be physically active (*p* = 0.018).

Regarding the intra-subject level, there are significant differences for the Time factor for the variable intention to be physically active (*p* = 0.002), and in the Time and Group interaction at the level of interpersonal style of autonomy support (*p* = 0.025). In the interaction between Time and Age there are significant differences for the intention to be physically active (*p* = 0.003).

In the pairwise comparison, there are significant differences between groups for the variable's satisfaction of the basic need for relationship (*p* = 0.007) and fun (*p* = 0.026). Over time there are significant differences for the relationship variable (*p* = 0.041). For the Group and Sex interaction in the autonomy support variable (*p* = 0.003), for competence (*p* = 0.039), for relationships (*p* = 0.003), for autonomous motivation (*p* = 0.017), for IPM (*p* = 0.024), for the intention to be physically active (*p* = 0.005) and for fun (*p* = 0.031), in all these cases always for the male sex and with higher values for the experimental group.

Given that there are interactions between the time and group factors for many of the variables, it was considered convenient to analyze the differences between the control and experimental groups for the pre-test and post-test separately. In the pre-test there are no significant differences between the control and experimental groups except for the satisfaction of the basic need for relationship (*p* = 0.003) in favor of the experimental group, which indicates that the groups are homogeneous with respect to the variables of interest, except for the last one mentioned. However, there are significant differences in the post-test in favor of the EG, and at the level of autonomy support (*p* = 0.006) and autonomous motivation (*p* = 0.031; Table 3).

**Table 3:** Multivariate analysis.

	**Cluster**	**Pre-test**		**Post-test**		**Pre-post comparison**	
		**Mean**	**SD**	**Mean**	**SD**	***p*-value**	**Diff (DT)**
Autonomy support	Control	4.341	0.162	3.990	0.174	0.116	0.351 (0.219)
Experimental	4.339	0.076	4.559	0.081	0.044^*^	−0.211 (0.102)
*p*-value + DT	0.989	0.180	0.006^**^	0.193		
Competence	Control	4.873	0.251	5.307	0.203	0.174	−0.433 (0.314)
Experimental	5.177	0.117	5.417	0.095	0.109	−0.239 (0.146)
*p*-value + DT	0.281	0.278	0.628	0.225		
Autonomy	Control	3.609	0.321	3.646	0.387	0.932	−0.037 (0.429)
Experimental	3.768	0.149	3.604	0.180	0.164	−1.235 (0.200)
*p*-value + DT	0.657	0.356	0.923	0.251		
Relationship	Control	4.111	0.226	4.680	0.270	0.083	−0.570 (0.322)
Experimental	4.901	0.105	5.075	0.126	0.251	−0.174 (0.150)
*p*-value + DT	0.003^**^	0.251	0.195	0.300		
IPM	Control	4.198	0.203	4.544	0.192	0.169	−0.347 (0.248)
Experimental	4.527	0.079	4.699	0.090	0.475	−0.083 (0.116)
*p*-value + DT	0.071	0.225	0.473	0.213		
Autonomous motivation	Control	6.330	0.207	6.413	0.125	0.744	−0.082 (0.251)
Experimental	6.337	0.097	6.721	0.058	0.002^**^	−0.384 (0.117)
*p*-value + DT	0.976	0.230	0.031^*^	0.138		
SDI	Control	14.104	2.053	14.990	1.795	0.692	−0.885 (2.222)
Experimental	13.424	0.956	16.486	0.836	0.005^**^	−3.062 (1.035)
*p*-value + DT	0.766	2.277	0.456	1.990		
IPA	Control	4.294	0.170	4.455	0.169	0.439	−0.160 (0.206)
Experimental	4.527	0.079	4.720	0.079	0.049^*^	−0.193 (0.096)
*p*-value + DT	0.225	0.189	0.164	0.188		
Fun	Control	3.693	0.145	3.739	0.101	0.777	−0.047 (0.164)
Experimental	3.956	0.067	3.958	0.047	0.984	−0.001 (0.076)
*p*-value + DT	0.108	0.160	0.058	0.112		

SDI, self-determination index; IPM, index of psychological mediators; IPA, Intention to be physically active; SD, Standard deviation, Dif, Difference of means.

^*^p < 0.05.

^**^p < 0.01.

On the other hand, if we compare the variables between the pre-test and the post-test for each group, for the CG there are no significant differences over time. On the other hand, for the EG the scores increased significantly for the variable's autonomy support (*p* = 0.044), self-determination index (*p* = 0.005), autonomous motivation (*p* = 0.002) and for the intention to be physically active (*p* = 0.049; Table 3).

**Figure 2:**
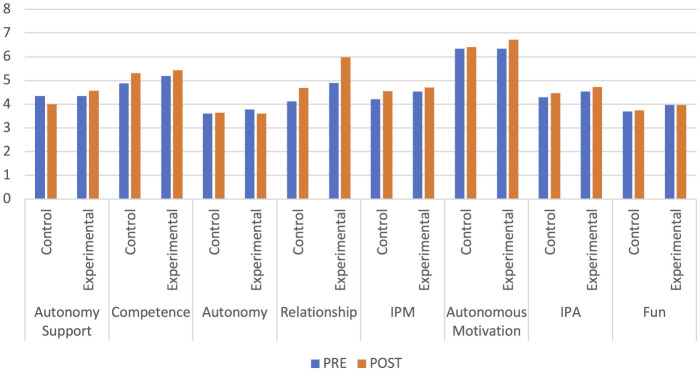
Pairwise comparisons between groups as a function of time. IPA, intention to be physically active; IPM, index of psychological mediators.

Regarding the differences at the sex level over time, significant differences are only found between men for the variable satisfaction of competence (*p* = 0.046), relationship (*p* = 0.032), IPM (*p* = 0.045) and intention to be physically active (*p* = 0.049). In the Group and Sex interaction, there are differences between sex for the control group in the interpersonal style variables of autonomy support (*p* = 0.039), and in the intention to be physically active (*p* = 0.030) with higher values in women. For the experimental group in the variables satisfaction of the psychological need for competition, with higher values in men (*p* = 0.006). For the Time and Sex interaction, in the pre-test there are significant differences for the interpersonal style of autonomy support (*p* = 0.032), and for the intention to be physically active (*p* = 0.049) with higher values for women. All these outcomes are represented in the Figure 2 with the means of each variable before and after the intervention and for control and experimental group.

Regarding the significant differences at the sex level within each group, they are found within the control group in the pre-test for the interpersonal style of autonomy support (*p* = 0.006) and in the self-determination index (*p* = 0.044). and in the intention to be physically active (*p* = 0.007), with higher values for men. For its part, in the experimental group there are only significant differences in the variable satisfaction of the psychological need for competence both in the pre-test (*p* = 0.049) and in the post-test (*p* = 0.030) in favor of men.

Finally, in the analysis of the Group, Sex and Time interaction, it is not found that there are differences in the experimental group over time for the male sex. At the experimental group level for the female sex, there are significant differences for the self-determination index (*p* = 0.008), autonomous motivation (*p* = 0.004) and the intention to be physically active (*p* = 0.029) with higher values in the post-test. Regarding the control group over time for the male sex, no difference was found. And for the female sex in support for autonomy (*p* = 0.024), presenting higher values in the pre-test.

When this analysis focuses on analyzing the Group, Sex and Time interaction between groups, significant differences are found for the male sex in the pre-test for the interpersonal style variables of autonomy support (*p* = 0.034), competence (*p* = 0.040), relationship (*p* < 0.001), IPM (*p* = 0.010), intention to be physically active (*p* = 0.003) with higher values for the experimental group (Table 4). And for the male sex in the post-test in the interpersonal style variables of autonomy support (*p* = 0.014) and autonomous motivation (*p* = 0.044), the experimental group presenting higher values. At the female level, significant differences are found in the pre-test for the interpersonal style variable of autonomy support (*p* = 0.037) with higher values for the control group.

**Table 4:** Pre post-test differences according to sex and group.

		**Pre-test**		**Post-test**		**Pre post-test differences**	
		**Male**	**Female**	**Male**	**Female**	**Male**	**Female**
		**Mean (SD)**	**Mean (SD)**	**Mean (SD)**	**Mean (SD)**	***p*-value**	***p*-value**
Autonomy support	Control	3.862 (0.224)	4.820 (0.240)	3.913 (0.240)	4.067 (0.257)	0.866	0.024^*^
Experimental	4.416 (0.113)	4.261 (0.100)	4.610 (0.122)	4.490 (0.107)	0.210	0.097
*p*-value	0.034^*^	0.037^*^	0.014^*^	0.136		
Competence	Control	4.586 (0.346)	5.161 (0.371)	5.352 (0.280)	5.262 (0.300)	0.083	0.828
Experimental	5.413 (0.175)	4.941 (0.154)	5.628 (0.142)	5.205 (0.125)	0.332	0.179
*p*-value	0.040^*^	0.588	0.388	0.863		
Autonomy	Control	3.249 (0.441)	3.969 (0.474)	3.658 (0.532)	3.634 (0.571)	0.492	0.599
Experimental	3.722 (0.224)	3.814 (0.197)	3.590 (0.270)	3.618 (0.238)	0 663	0.451
*p*-value	0.354	0.764	0.911	0.980		
Relationship	Control	3.720 (0.311)	4.502 (0.334)	4.584 (0.372)	4.777 (0.399)	0.057	0.566
Experimental	4.975 (0.158)	4.827 (0.139)	5.195 (0.189)	4.955 (0.166)	0.331	0.522
*p*-value	< 0.001^**^	0.374	0.154	0.682		
IPM	Control	3.852 (0.280)	4.544 (0.300)	4.531 (0.265)	4.557 (0.284)	0.053	0.971
Experimental	4.703 (0.142)	4.527 (0.125)	4.805 (0.134)	4.593 (0.118)	0.561	0.671
*p*-value	0.010^*^	0.960	0.367	0.909		
Autonomous motivation	Control	5.963 (0.285)	6.697 (0.306)	6.362 (0.171)	6.463 (0.184)	0.253	0.530
Experimental	6.466 (0.145)	6.209 (0.128)	6.765 (0.087)	6.677 (0.027)	0.094	0.004^**^
*p*-value	0.127	0.148	0.044^*^	0.289		
SDI	Control	9.765 (2.826)	18.444 (3.03)	13.913 (2.47)	16.066 (2.65)	0.182	0.472
Experimental	13.237 (1.43)	13.611 (1.26)	15.561 (1.25)	17.411 (1.10)	0.141	0.008^**^
*p*-value	0.284	0.148	0.559	0.644		
IPA	Control	3.800 (0.234)	4.789 (0.252)	4.332 (0.233)	4.740 (0.118)	0.067	0.491
Experimental	4.638 (0.119)	4.416 (0.105)	4.740 (0.118)	4.700 (0.104)	0.479	0.029^*^
*p*-value	0.003^**^	0.178	0.129	0.653		
Fun	Control	3.610 (0.199)	3.775 (0.214)	3.762 (0.139)	3.717 (0.150)	0.503	0.809
Experimental	4.025 (0.101)	3.887 (0.089)	4.002 (0.071)	3.913 (0.062)	0.836	0.791
*p*-value	0.072	0.632	0.137	0.162		

SDI, self-determination index; IPM, index of psychological mediators; IPA, Intention to be physically active; SD, Standard deviation.

^*^p < 0.05.

^**^p < 0.01.

### 3.3 Interview results

Continuing with the analysis of the interviews, four themes (codes) were obtained with a total of 24 extracts (quotes). These excerpts were collected throughout the three questions regarding the coaches' knowledge of the PBA and the applicability of the PBA.

The results were classified into three groups depending on whether they were positive, neutral or negative aspects of the PBA. The group of positive aspects was made up of the codes called “fun and motivation.” The group of neutral aspects was made up of the codes “interest in the activity”. And finally, the group of negative aspects was made up of the code “play activities weaknesses.”

The two codes that stand out the most for their foundation belong to the group of so-called positive aspects, with the code “fun” made up of 10 extracts and with comments such as “When I carry out the activities of this methodology, I have a lot of fun” (athlete A, federation B). As for the “motivation” code, it refers to the perception that children have of the different play activities, being the third most mentioned code with six excerpts, with statements such as those of athlete C, Federation A who indicates “the coach is giving us motivation and for us it is important.”

The second group was made up of the code “interest in the activity,” linked to the importance of the play activities in the warm-up, this being the main objective of the coaches. This code was made up of five extracts, where, for example, participant D, federation A stated, “long distance relay play forms are important because they are activities to improve our physical capabilities.”

Finally, the third group was made up of a single code, called weaknesses of the play activities with three extracts related to the participants, as also clarified by interviewee C, Federation B, “we carry out activities different from those we know and I am worried if I will be ready for my training today.”

Analyzing the interviews with the athletes, it can be seen that the application of this methodology for the first time in their training has a positive effect and acceptance by the experimental group of athletes, since it is evident that they like it, it seems new to them and it motivates them to actively continue their sports preparation, in this way it is appreciated that they find it non-repetitive. Furthermore, there is an interest in the activities because they are concerned with improving their physical condition. Finally, they are concerned about being fit for the main part of the training.

## 4 Discussion

The present study had two main objectives. On the one hand, analyze the effects of the PBA on different psychological variables following the self-determination theory and the possible differences depending on sex. On the other hand, to discover the perceptions of athletes about the PBA and its usefulness in provincial sports federations in the athletics discipline.

Regarding the first objective of finding out the effects of the PBA on different psychological variables, the PBA participants presented an improvement in the interpersonal style of the coach of autonomy support and in autonomous motivation compared to the control group, which coincides with the study by Yupa-Pintado and Heredia-León (2021) in young athletes between 9 and 18 years old, where a PBA was also applied in athletics schools for 12 weeks with 2 weekly sessions. This study, in addition to showing an improvement in the three psychological needs satisfaction, also did so in self-determined motivation. These results partly coincide with those obtained in the present investigation, where the athletes participating in the PBA presented improvements in autonomous motivation, along with a higher rate of self-determination and intention to continue practicing athletics. Blatsis et al. (2016), in a study with more than 200 children between the ages of 11 and 12, implemented a program based on the PBA for 12 weeks, finding significant improvements in more self-determined motivation and the intention to continue practicing athletics against the conventional teaching group, as well as improvements in physical condition variables. These results coincide with those obtained in the present study, where autonomous motivation and the intention to continue practicing athletics were higher in the post-test for the group that followed the PBA, in addition to the interpersonal style of the coach and the self-determination index.

In line with the above, Valero-Valenzuela et al. (2009), in a descriptive study with around 250 young Spanish athletes, reported high and positive correlations between the interpersonal style of the coach who provided autonomy to his athletes and their intention to continue practicing athletics. Later Heredia-León et al. (2023), in a predictive study with young Ecuadorian athletes, confirmed the predictive capacity that the interpersonal style of autonomy support had on the satisfaction of basic psychological needs, which positively predicted autonomous motivation and, as a result, the intention to be physically active athletes, in line with the self-determination theory of Ryan and Deci (2000).

Other studies, within the educational field of primary education, such as the work of Peraza et al. (2018) was carried out with a sample of 5th grade students and 6th grades between 10 and 12 years of age, from two primary schools, focusing their attention on the teaching-learning process of athletics. They implemented a set of alternative activities to motivate the teaching of athletics from the earliest ages in a playful way, finding that after the application of the program, motivation increased significantly in the sample of athletes. Similarly, Navarro Patón et al. (2018), who used competitive play activities in athletics during a six-session teaching unit, also found significant improvements in intrinsic motivation and in the satisfaction of the basic psychological needs of competition and relationships with others.

Regarding fun, this work coincides with the results obtained by Navarro Patón et al. (2018), who used competitive athletics play forms for six sessions without obtaining significant improvements in enjoyment compared to the control group. On the other hand, this work presents discrepancies with the results of other studies such as that of Sánchez-Morales et al. (2016) or that of Valero-Valenzuela et al. (2012), who do report higher levels of fun, although in these cases, like the study by Navarro Patón et al. (2018), the PBA was applied in a primary and secondary school context.

It is worth noting that there were differences at the sex level. In the PBA group, male athletes obtained higher values compared to the conventional group in the interpersonal style variables of autonomy support and autonomous motivation. These results are in line with those found by Navarro Patón et al. (2018), who reported greater improvements in boys in the variables of intrinsic motivation, competition and enjoyment. These data may be due to the fact that the proposals were very focused on competition and therefore generated greater social pressure on women compared to men Goñi-Grandmontagne et al. (2004). Regarding the differences over time, the women in the control group presented a greater perception of the coach's interpersonal style of autonomy support in the pre-test, while the women in the experimental group showed higher values in the post-test in autonomous motivation, self-determination index and intention to continue practicing athletics. These findings could be related to different variables such as cultural or social aspects, as well as aspects related to training intensities or expectations, which might partially explain the higher values of autonomous motivation of women.

Addressing the second objective of this study, which was to find out the perceptions that athletes had about the use of the PBA. The results indicate that their vision is very good, like Yupa-Pintado and Heredia-León (2021) with athletes in an athletics school, where it is stated that the Ludotechnical Model, which uses played forms and facilitates a set of rules for them gradually to acquire the technique (Pizarro et al., 2024), caused positive results in training and good acceptance by athletes, and in turn the athletes perceived it as novel and fun in their training. In turn, Valero-Valenzuela et al. (2009) although within the school environment, indicate that students perceive the proposal as fun, but on the other hand, the teachers who apply it feel that learning is not achieved. Results that are in line with the findings in the present study, since it focused on the opinion of young people who are concerned about improving their fitness and are expecting to be trained. This could certainly be the case here since it is a sample of federated athletes, they are more demanding in terms of worrying about obtaining results, compared to the sample of schoolchildren.

The time dedicated to the implementation of the PBA only in the specific part of the warm-up was limited. The competitions scheduled during the weekends, the ages of the athletes and belonging to a specialization group made it difficult to achieve longer intervention times, although on the other hand, this type of implementation may be the most realistic for application in federations and athletics clubs that have athletes who are no longer in the sports initiation stage. The proximity of the National Championship of Ecuador prevented the intervention program from being prolonged, an aspect that would be interesting to consider in future studies. However, these data cannot be generalized, since the present study is subject to some limitations such as the sample, which is a non-probabilistic and non-representative sample. Moreover, the use of self-reported data could introduce biases or limit generalizability therefore, we must be very cautious, reproducing similar studies in other populations to be able to consider that it is a generalizable fact. Furthermore, the absence of randomization and lack of control for possible contaminating variables may have introduced biases that have influenced the results obtained. These limitations should be considered for future studies. Additionally, it is recommended to measure other variables linked to performance on the event they take and the effects of this type of approach on their results.

## 5 Conclusions

The study revealed that the PBA induced beneficial effects on the athletes' perception of the EAA, on autonomous motivation and on the intention to continue practicing athletics, especially in women over time. The perception of young people is an increase in fun, interest and motivation toward the activity, although at the same time also concern about the rest of the training session. In light of these findings, it is suggested that athletic trainers consider employing playful pedagogical strategies such as PBA in athletics training sessions, given their ability to foster a coach motivational style that supports athlete autonomy, and its potential positive effects on motivation and intention to continue their practice in the future. Moreover, integrating play-based approaches into various athletic disciplines, beyond regional-level training could yield relevant and specific information depending on the sport context.

## Data Availability

The raw data supporting the conclusions of this article will be made available by the authors, without undue reservation.

## References

[B1] Aguirre A. (1995). Qualitative methodology in sociocultural research. Ethnography. Barcelona: Marcombo.

[B2] Atiq A. Tangkudung J. Mulyana G. (2017). Development of BASIC techniques procurement model for soccer athletes based on play for beginners ages 8-12 years. J Indonesian Phys. Educ. Sport. 3, 110–121. 10.21009/JIPES.032.09

[B3] Balaguer I. Atienza F. L. Castillo I. Moreno Y. Duda J. L. (1997). “Factorial structure of measures of satisfaction/interest in sport and classroom in the case of Spanish teenagers,” in Proceedings of the Abstracts of Fourth European Conference of Psychological Assessment (Lisbon, Portugal), 76.

[B4] Blanchard K. Cheska A. (1986). Anthropology of Sport. Bellaterra: Barcelona, Ediciones SA.

[B5] Blatsis P. Saraslanidis P. Barkoukis V. Manou V. Tzavidas K. Palla S.. (2016). The effect of IAAF kids athletics on the physical fitness and motivation of elementary school students in track and field. J. Phys. Educ. Sport. 16, 883–896. 10.7752/jpes.2016.0313933809896

[B6] Böke H. Aygün Y. (2024). Effects of tactical game model on multidimensional developmental domains: a systematic review and meta-analysis. Pedagogical News. 82:e1775. 10.19052/ap.vol1.iss82.7

[B7] Bubikova-Moan J. Hjetland N. Wollscheid S. E. C. E. (2019). teachers' views on play-based learning: a systematic review. Eur. Early Child Educ. Res. J. 27, 776–800. 10.1080/1350293X.2019.1678717

[B8] Coakley J. (2021). Sports in Society: Issues and Controversies, 13th Edn. London: McGraw-Hill.

[B9] Curran P. J. West S. G. Finch J. F. (1996). The robustness of test statistics to nonnormality and specification error in confirmatory factor analysis. Psychol. Methods. 1:16. 10.1037/1082-989X.1.1.1626174714

[B10] Danniels E. Pyle A. (2018). “Defining play-based learning,” in Encyclopedia on Early Childhood Development, eds. R. E. Tremblay, M. Boivin, R. D. V. Peters, and A. Pyle. Available at: https://www.child-encyclopedia.com/play-based-learning/according-experts/defining-play-based-learning (accessed December 6, 2024).

[B11] Díaz-Varela A. Wright L. (2019). Play for adults: play-based approaches in teacher training. Scott. Educ. Rev. 51, 132–136. 10.51166/ser/512diazvarela

[B12] Duda J. L. Nicholls J. G. (1992). Dimensions of achievement motivation in schoolwork and sport. J. Educ. Psychol. 84, 290–299. 10.1037/0022-0663.84.3.290

[B13] Gabbett T. J. Jenkins D. Abernethy B. (2009). Game-based training for improving skill and physical fitness in team sport athletes. Int. J. Sports SciCoach. 4, 273–283. 10.1260/17479540978854955329486750

[B14] Gambles E. Gutierrez D. (2023). An international consensus on terminology: game-based vs game-centered. Int. Matters. Available at: https://sure.sunderland.ac.uk/id/eprint/17360/

[B15] García-López L. M. Gutiérrez Díaz del Campo D. González-Víllora S. Valero Valenzuela A. (2012). Changes in empathy, assertiveness and social relationships due to the application of the sports education instruction model. Rev. Psychol. Sport 21, 321–330.

[B16] Goñi-Grandmontagne A. Ruiz de Azúa-García S. Rodríguez-Fernández A. (2004). Sports and physical self-concept in preadolescence. Notes Phys. Educ. Sports 3, 18–24. Available at: https://raco.cat/index.php/ApuntsEFD/article/view/301451

[B17] Hastie P. Casey A. (2014). Fidelity in models-based practice research in sport pedagogy: a guide for future investigations. J. Teach. Phys. Educ. 33, 422–431. 10.1123/jtpe.2013-0141

[B18] Hein V. Müür M. Koka A. (2004). Intention to be physically active after school graduation and its relationship to three types of intrinsic motivation. Eur. Phys. Educ. Rev. 10, 5–19. 10.1177/1356336X04040618

[B19] Heredia-León D. A. Manzano-Sánchez D. Gómez-Mármol A. Valero-Valenzuela A. (2023). Prediction of the adherence to sports practice of young Ecuadorians based on the perception of the coach's interpersonal style. Front. Psychol. 14:1133583. 10.3389/fpsyg.2023.1133583 37179851 PMC10169726

[B20] Hoyos L. (2024). “Sport as a scientific discipline and its teachability,” in Science and Mathematics Education: Contexts, Challenges and Opportunities, eds. L. Parga, P. Zapata, and R. Tuay-Sigua (Bogotá: UPN Editorial), 423–428.

[B21] Kerlinger F. C. Howard L. E. (2022). Behavioral Research. New York, NY: McGraw-Hill.

[B22] Kinnerk P. Harvey S. MacDoncha C. Lyons M. (2018). A review of the game-based approaches to coaching literature in competitive team sport settings. Quest 70, 401–419. 10.1080/00336297.2018.1439390

[B23] Lonsdale C. Hodge K. Rose E. A. (2008). The behavioral regulation in sport questionnaire (BRSQ): instrument development and initial validity evidence. J. Sport Exerc. Psychol. 30, 323–355. 10.1123/jsep.30.3.323 18648109

[B24] Manzano-Sánchez D. Postigo-Perez L. Gomez-Lopez M. Valero-Valenzuela A. (2020). Study of the motivation of Spanish amateur runners based on training patterns and gender. J. Environ. Res. Public Health 17:8185. 10.3390/ijerph17218185 33167506 PMC7663920

[B25] Manzano-Sánchez D. Valero-Valenzuela A. Conde-Sanchez A. Ming-Yao C. (2019). Applying the personal and social responsibility model-based program: differences according to gender between basic psychological needs, motivation, life satisfaction and intention to be physically active. J. Environ. Res. Public Health 16:2326. 10.3390/ijerph16132326 31266245 PMC6651884

[B26] Mitchell S. Oslin J. Griffin L. (2020). Teaching Sport Concepts and Skills: A Tactical Games Approach. Champaign, IL: Human Kinetics.

[B27] Moreno J. A. Moreno R. Cervelló E. (2007). The physical self -concept as predictor of the intention of being physically active. Psychol. Health 17, 261–267.

[B28] Moreno-Murcia J. Huéscar E. Andrés-Fabra J. Sánchez-Latorre F. (2020). Adaptation and validation of autonomy support and controller style's scales in physical education: relationship with feedback. Rev. Sci. Act Phys. 21, 1–16. 10.29035/rcaf.21.1.3

[B29] Moreno-Murcia J. A. Marzo J. C. Martínez-Galindo C. Conte L. (2011). Validation of psychological need satisfaction in exercise scale and the behavioral regulation in sport questionnaire to the Spanish context. Rev. Int. Hundred Dep. 7, 355–369. 10.5232/ricyde2011.02602

[B30] Navarro Patón R. Cons-Ferreiro M. Eirín Nemiña R. (2018). Effect of a teaching unit based on competitive games on motivation, basic psychological needs and enjoyment in Primary Education students. Sports 4, 111–125. 10.17979/sportis.2018.4.1.2900

[B31] Ntshangas N. Venketsamy R. (2022). Practitioners' perceptions of play-based pedagogy on the holistic development of young children. Profesi Pendidikan Dasar. 9, 149–162. 10.23917/ppd.v9i2.18477

[B32] Nunnally J. C. (1978). Psychometric Theory. New York, NY: McGraw-Hill.

[B33] O'Connor D. Larkin P. Höner O. (2020). “Coaches' use of game-based approaches in team sports,” in Perspectives on Game-Based Coaching (London: Routledge). 10.4324/9781003007272-12

[B34] Peraza C. Morales C. Rodríguez M. (2018). Games to motivate the teaching of athletics at an early age from the Physical Education class. Podium 13, 287–300.

[B35] Pérez-González A. Valero-Valenzuela A. Moreno-Murcia J. Sánchez-Alcaraz B. (2019). Systematic review of autonomy support in physical education. Notes Educ. Phys. Esports 35, 51–61. 10.5672/apunts.2014-0983.cat.(2019/4).138.04

[B36] Pizarro D. Cosín J. González-Cutre D. González-Fernández F. T. Práxedes A. (2024). Influence of ludotechnical model and teaching games for understanding on roller hockey player motivation. Apunts. Educació Física i Esports 157, 31–39. 10.5672/apunts.2014-0983.es.(2024/3).157.04

[B37] Pyle A. Danniels E. (2017). A continuum of play-based learning: the role of the teacher in play-based pedagogy and the fear of hijacking play. Early Educ. Dev. 28, 274–289. 10.1080/10409289.2016.1220771

[B38] Ribas J. Hernandez-Moreno J. Diaz-Diaz R. Borges-Hernandez P. Ruiz-Omeñaca J. Jaqueira A. (2023). How to understand sports and traditional games and how to apply Item to physical education. On the “Goal of Game”. Sports Act Living. 5:1123340. 10.3389/fspor.2023.1123340 36926620 PMC10011656

[B39] Ryan R. M. Deci E. L. (2000). Intrinsic and extrinsic motivations: classic definitions and new directions. Contemp. Educ. Psychol. 25, 54–67. 10.1006/ceps.1999.1020 10620381

[B40] Sánchez-Morales M. Valero-Valenzuela A. Manzano-Sánchez D. López-Jiménez J. (2016). Effects of a ludotechnical teaching unit on the learning of high jump by high school students, Ágora PE Sport 2, 199–213.37555441

[B41] Seale C. (1999). The Quality of Qualitative Research. London: SAGE Publications. 10.4135/9780857020093

[B42] Sturmey P. Newton J. T. Cowley A. Bouras N. Holt G. (2005). The PAS–ADD checklist: independent replication of its psychometric properties in a community sample. Br. J. Psychiatry. 186, 319–323. 10.1192/bjp.186.4.319 15802689

[B43] Taylor S. J. Bogdan R. (1992). Introduction to Qualitative Research Methods Barcelona: Paidós.

[B44] Thyer B. A. (2012). Quasi-Experimental Research Designs. Oxford: Oxford University Press. 10.1093/acprof:oso/9780195387384.001.0001

[B45] Triansyah A. Haetami M. Hidasari F. (2019). Athletic learning support games for junior high school students. procedures of the 1st international conference on sport sciences, health and tourism. Adv. Health Sci. Res. 35, 162–167. 10.2991/ahsr.k.210130.03332175718

[B46] Valero-Valenzuela A. Conde-Sánchez A. Delgado-Fernández M. Conde- Caveda J. L, De la Cruz-Sánchez, E. (2012). Effects of traditional and ludotechnical instructional approaches on the development of athletics performance, efficiency and enjoyment. Didact. Slov. 3−4, 51–66.

[B47] Valero-Valenzuela A. Delgado M. Conde J. L. (2009). Motivation towards the practice of athletics in primary education based on two teaching/learning proposals. J. Sports Psychol. 18, 123–136.27409075

[B48] Valero-Valenzuela A. Huescar E. Núñez J. L. Conte L. Leon J. Moreno-Murcia J. A.. (2021). Prediction of adolescent physical self-concept through autonomous motivation and basic psychological needs in Spanish. Phys. Educ. Stud. Sustain. 13:11759. 10.3390/su13211175927409075

[B49] Williams G. C. Grow M. V. Feedman R. Z. Ryan R. M. Deci E. L. (1996). Motivational predictors of weight loss and weight-loss maintenance. J. Pers. Soc. Psychol. 70, 115–126. 10.1037/0022-3514.70.1.115 8558405

[B50] Wilson P. Rogers W. Rodgers W. Wild T. C. (2006). The psychological need satisfaction in exercise scale. J. Sport Exerc. Psychol. 28, 231–251. 10.1123/jsep.28.3.231

[B51] Wright P. Craig M. (2011). Tool for assessing responsibility-based education (TARE): instrument development, content validity, and inter-rater reliability. Meas. Phys. Educ. Exerc. Sci. 15, 204–219. 10.1080/1091367X.2011.590084

[B52] Yupa-Pintado E. X. Heredia-León D. A. (2021). Incidence of the ludotechnical model on motivation in the practice of athletics. KOINONIA Interdiscip. Refereed J. 6, 707–733. 10.35381/r.k.v6i2.1277

